# Laboratory Manual of Physiology

**Published:** 1906-04

**Authors:** 


### BOOKS AND AUTHORS.

**Laboratory Manual of Physiology.** By **Frederick C. Busch, B.S.,** M.D., Professor of Physiology, Medical Department, University of Buffalo. Illustrated. Small Octavo, pp. 206. New York: William Wood & Co., 1905. (Price, $1.25).

It is pleasant to find upon the title page of this volume the name of a Buffalo physician—that of Dr. Frederick C. Busch, well known as a teacher given to research and as one who has gained success in his special calling. The views of the author are presented with singular clearness, and his work forms an admirable substitute for the ordinary outlines, or lesson sheets, employed in so many laboratories. The subject in hand is treated in a concise and comprehensive way, which is not so easily done when giving an outline of experiments in the various departments of physiology, particularly in those which are of the most practical value to the student.

Dr. Busch has successfully accomplished the difficult task of compressing into a manual of moderate size the enormous mass of facts which pertain to our present methods of physiological teaching, as connected with training in technic, and as the basis of its application to actual medical problems, which will do more toward making the student an intelligent practitioner than all former methods. The student, under the directions of this laboratory guide, is to work out for himself the physiological problems given by the manual, aided by original illustrations. The subject matter includes muscle-nerve physiology, nervous system, blood circulation, respiration, secretion, digestion, absorption, internal secretion and excretion, and general special sensations.

Each chapter is entitled to consideration for its peculiar features and important suggestions. The chapter on Internal Secretion is, perhaps, the one most worthy of special mention, since it treats of matters not usually included in a physiological course.

The author is to be congratulated upon the excellent way in which he has accomplished his task. His conclusions throughout are based upon his extensive personal experience, and it is this which gives the keynote to the special value of this volume. The mechanical construction of the book also deserves praise.

